# Design and simulation of a PLC-controlled omni wheel conveyor sorting system for high-speed material handling

**DOI:** 10.1038/s41598-025-13164-3

**Published:** 2025-10-01

**Authors:** Ritesh Bhat, Shilpa Suresh, D. Devakumar, K. R. Subramanian, D. Dhanush, Subramaniam Krishnan

**Affiliations:** 1 Department of Mechatronics Engineering, Rajalakshmi Engineering College, Thandalam, Tamil Nadu 602105 India; 2 Department of Mechatronics, Manipal Institute of Technology, Manipal Academy of Higher Education, Manipal, India; 3 Department of Mechanical Engineering, MILA University, 71800 Nilai, Negeri Sembilan Malaysia

**Keywords:** Omni wheel sorting, Programmable logic controllers, Material handling, Automation, Virtual commissioning, Electrical and electronic engineering, Mechanical engineering

## Abstract

This study proposes a PLC-controlled omni wheel conveyor sorting system designed to address limitations in traditional sorting mechanisms by integrating barcode-based classification with high-speed and adaptable sorting capabilities. The system utilizes a Siemens S7-1200 PLC, omni wheels, a barcode scanner, and a conveyor motor to achieve precise, flexible, and efficient material handling. Mathematical analysis validated the system’s structural integrity, with deflections under $0.009 \, \text {mm}$, and operational efficiency, including a roller speed of $35.46 \, \text {RPM}$ to support a throughput of 2000 objects per hour at a conveyor speed of $0.167 \, \text {m/s}$. Simulations conducted in Factory IO achieved a sorting accuracy of $98\%$, demonstrating the seamless synchronization of barcode scanning and sorting operations through deterministic ladder logic. The omni wheels provided multidirectional flexibility, reducing energy losses and enabling rapid redirection of objects. Compared to reinforcement learning-based approaches, the proposed system offers simplicity, cost-effectiveness, and ease of implementation without compromising accuracy or scalability. However, the simulations assumed ideal conditions, and limitations such as environmental factors, dynamic loading, and real-world scalability remain unaddressed. Future research could explore integrating IoT-enabled monitoring, hybrid control strategies, and dynamic adaptability to enhance performance in complex industrial environments. The results highlight the potential of this system to revolutionize material handling across manufacturing, logistics, and e-commerce sectors.

## Introduction

Material handling (MH) systems constitute the cornerstone of industrial operations, ensuring the seamless movement of materials through various stages of production, assembly, and distribution^1–4^. Issues such as mechanical wear, design rigidity and inability to handle complex or high-throughput sorting requirements pose significant challenges in traditional sorting mechanisms^5^. These limitations underscore the need for innovative and adaptable solutions. In the context of modern manufacturing, where efficiency, precision, and scalability are essential to competitiveness, the adoption of automated systems has revolutionized operational workflows^6,7^. Among these advancements, automated sorting systems (ASS) have emerged as a critical enabler, streamlining material handling processes and addressing the growing demands of e-commerce, logistics, and manufacturing industries. These systems facilitate rapid processing, essential for next-day or same-day deliveries, and manage high shipment volumes, exemplified by the UPS air hub in Cologne, which sorts up to as much as 190,000 shipments per hour. Moreover, they enhance reliability by minimizing errors, protect ergonomic well-being by handling heavy and bulky items, and improve customer trust through consistent performance^8^. Such attributes make ASS indispensable in modern supply chains, driving efficiency and reliability across diverse industrial domains.

Programmable Logic Controllers (PLCs) have emerged as a robust alternative, offering programmable control, faster response times, and enhanced accuracy for sorting tasks. For example, Simran et al.^9^ developed an ASS that utilized PLC, incorporated with a conveyor belt, photoelectric sensors, ladder logic programming, and a Human-Machine Interface (HMI). This system demonstrated significant improvements in sorting accuracy, labor cost reduction, and error minimization, exemplifying the transformative potential of PLCs in automation. Kong et al.^10^ developed an automatic sorting system for wood diameter grades to improve efficiency and reduce costs in wood production.

The system used image-processing techniques to accurately measure the diameters of the wood, allowing automated classification into predefined categories. Experimental results demonstrated that the system significantly outperforms manual sorting methods in both speed and accuracy, meeting the demands of modern automated production environments. Gan et al.^11^ developed an automated cucumber inspection system that uses neural networks to improve precision agriculture and smart farming practices. The system integrates both software and hardware components, employing an industrial camera to capture images of cucumbers on a conveyor belt. It comprised three detection modules: cucumber identification, geometric property approximation, and defect detection. If a defect was detected, a Programmable Logic Controller (PLC) activated a motor to segregate the cucumber into an alternative container. Experimental evaluations on a dataset of 4,620 images demonstrated promising performance, with cucumber identification achieving an average Weighted Intersection over Union (WIoU) of 93%, volume approximation accuracy of 98%, and defect detection WIoU of 92%. The study suggested that this system can be integrated into online automatic sorting and grading processes to improve efficiency in agricultural production.

The versatility and adaptability of PLCs further extend their relevance in automation, particularly in educational and industrial domains, where user-friendly, cost-effective, and reliable solutions are paramount^12^. Zulfiqar et al.^13^ demonstrated a PLC-based sorting system capable of detecting and sorting objects by material type and size with remarkable precision and speed. Similarly, Vandana et al.^14^ designed a color-based sorting machine that uses a PLC as the master controller and an Arduino as the slave device, achieving high-speed and error-free sorting. Recent advances, such as the system proposed by Alaameri et al.^15^, integrate PLCs with advanced software platforms such as TIA Portal and Factory I/O. These sets of controls offer seamless control and monitoring, significantly enhancing productivity in manufacturing environments while reducing the reliance on manual labor.

Beyond traditional conveyor setups, the advent of omnidirectional conveyors marks a paradigm shift in material handling. Utilizing omni wheels, which enable smooth multidirectional movement, these systems cater to complex and space-constrained industrial environments^16,17^. Omni wheels, characterized by their innovative design with rollers attached perpendicularly to the base wheel’s axis, provide multidirectional mobility and enhanced flexibility. Zaher et al.^18^ further advanced this domain by integrating reinforcement learning (RL) algorithms such as Q-learning, double Q-learning, and deep Q-learning (DQN) for path planning and sorting tasks. Their approach eliminated the dependency on traditional mathematical models, enabling self-learning for optimal sorting mechanisms. Case studies demonstrated that RL-based methods outperformed traditional systems in convergence time and collision handling, emphasizing their potential for high-efficiency industrial applications.

Despite significant advancements in conveyor sorting systems as learnt from the discussed past studies, existing approaches often focus on specific functionalities, such as path planning, material type sorting, or modularity, without integrating these elements into a comprehensive solution. Although reinforcement learning and fuzzy logic methods enhance flexibility, they introduce computational complexity and scalability challenges, limiting their industrial applicability. Conversely, PLC-based systems offer simplicity and reliability, but are typically restricted to low-speed operations or predefined tasks, lacking the adaptability required for dynamic sorting environments. Furthermore, while barcode-based classification offers object-specific sorting advantages, its integration with flexible mechanisms such as omnidirectional wheels remains significantly underutilised. This underuse may stem from practical challenges in synchronising sensor data with real-time mechanical redirection, latency in scanning systems, or a general lack of modular PLC-compatible designs in current industrial setups. Additionally, most barcode-enabled systems are implemented in rigid path conveyors, not fully capitalising on the spatial adaptability provided by omni wheels. Existing studies validate systems through simulations or small-scale prototypes, but rarely address full-scale industrial implementation under realistic environmental conditions. This study aims to bridge these gaps by developing a PLC-controlled omni wheel conveyor sorting system that integrates barcode-based classification with high-speed and adaptable sorting, validated through high-throughput simulations in Factory I/O. The proposed system addresses the limitations of conventional PLC-based sorters by enabling real-time redirection of objects based on barcode data, while maintaining the deterministic reliability and low-cost advantages of PLC logic. In doing so, it provides a unified, scalable solution for future-ready industrial material handling applications.

## Methods

### Component selection

**Fig. 1:**
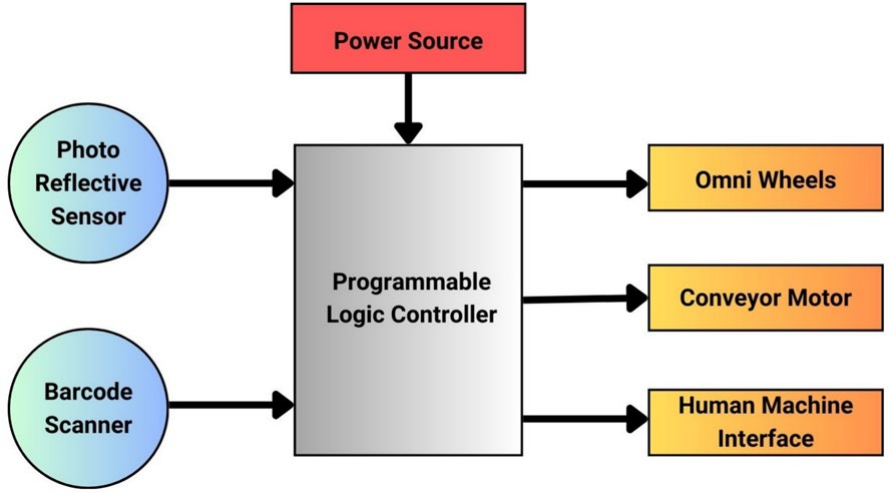
Proposed system architecture.

The system’s components were selected to ensure high speed, precision, and reliability while maintaining cost-effectiveness and compatibility with operational requirements. The Siemens S7-1200 PLC was chosen for its robust performance, ladder programming capabilities, and seamless integration with sensors and actuators. The Voyager XP1470G barcode scanner was selected to ensure accurate and high-speed object identification with minimal delays. Omni wheels were selected to provide multidirectional movement, allowing flexible and precise sorting, while their modular design supports dynamic path adjustments. The Bharat Bijlee IE2 Series AC motor was selected based on its industrial specifications, which made it the most suitable choice in terms of energy efficiency and operational reliability. The Siemens KTP700 Basic HMI was used to facilitate real-time monitoring and control, improving user interaction and troubleshooting. High-sensitivity photo-reflective sensors were employed to obtain reliability in detecting objects on the conveyor. Together, these components ensured that the system met the performance and efficiency goals. The architecture, as shown in Fig. 1, demonstrates the interconnections between these components.

### Proposed working principle

The photo-reflective sensor will detect objects on the conveyor belt and transmit data to the PLC, while the barcode scanner simultaneously reads and transmits classification information. The PLC will process this data to determine the sorting path and activate the omni wheels to direct objects to their designated destinations. The conveyor motor will then ensure consistent material flow, and the HMI will provide real-time status updates and control options. Programmed with ladder logic in the Siemens S7-1200 environment, the control sequence will begin with system initialization upon pressing the start button. The timers and counters in the ladder logic will synchronize object detection, data processing, and sorting operations.

### Design and analysis

**Fig. 2:**
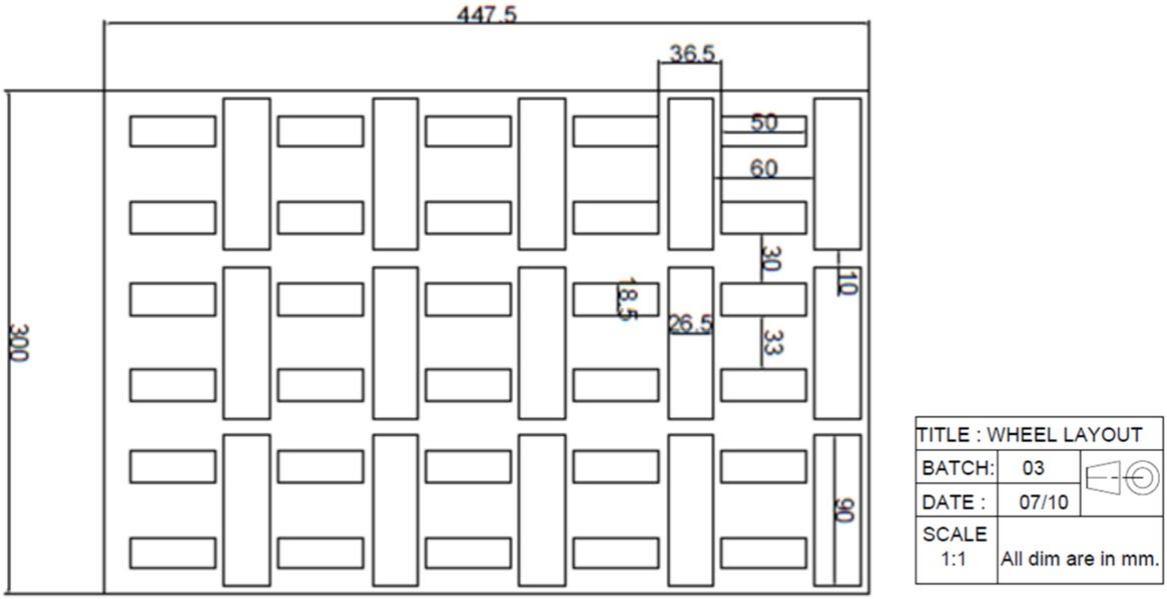
2D layout of the omni wheel conveyor system.

**Fig. 3:**
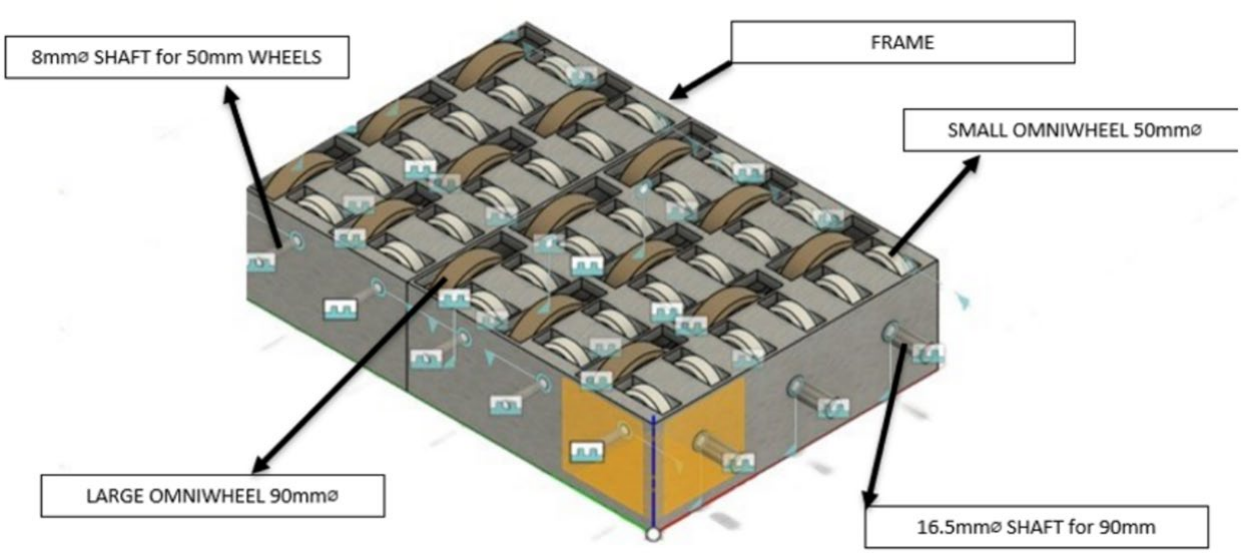
3D visualization of the wheel layout for the conveyor.

The 2D (Fig. 2) and 3D models (Fig. 3) of the omni wheel layout were developed to ensure an optimized design. The layout designed was to ensure the effective use of space while maintaining a smooth material flow.

The structural integrity of the conveyor frame was assessed using beam theory. Reactions on the supports were calculated using equilibrium equations, expressed as:

1 $$\begin{aligned} R_a + R_b = \sum F_y \end{aligned}$$

where $R_a$ and $R_b$ are reaction forces. The bending moment at any section $x$ is:

2 $$\begin{aligned} M(x) = R_a x - \sum F \cdot (x - \text {distance to load}) \end{aligned}$$

**Fig. 4:**
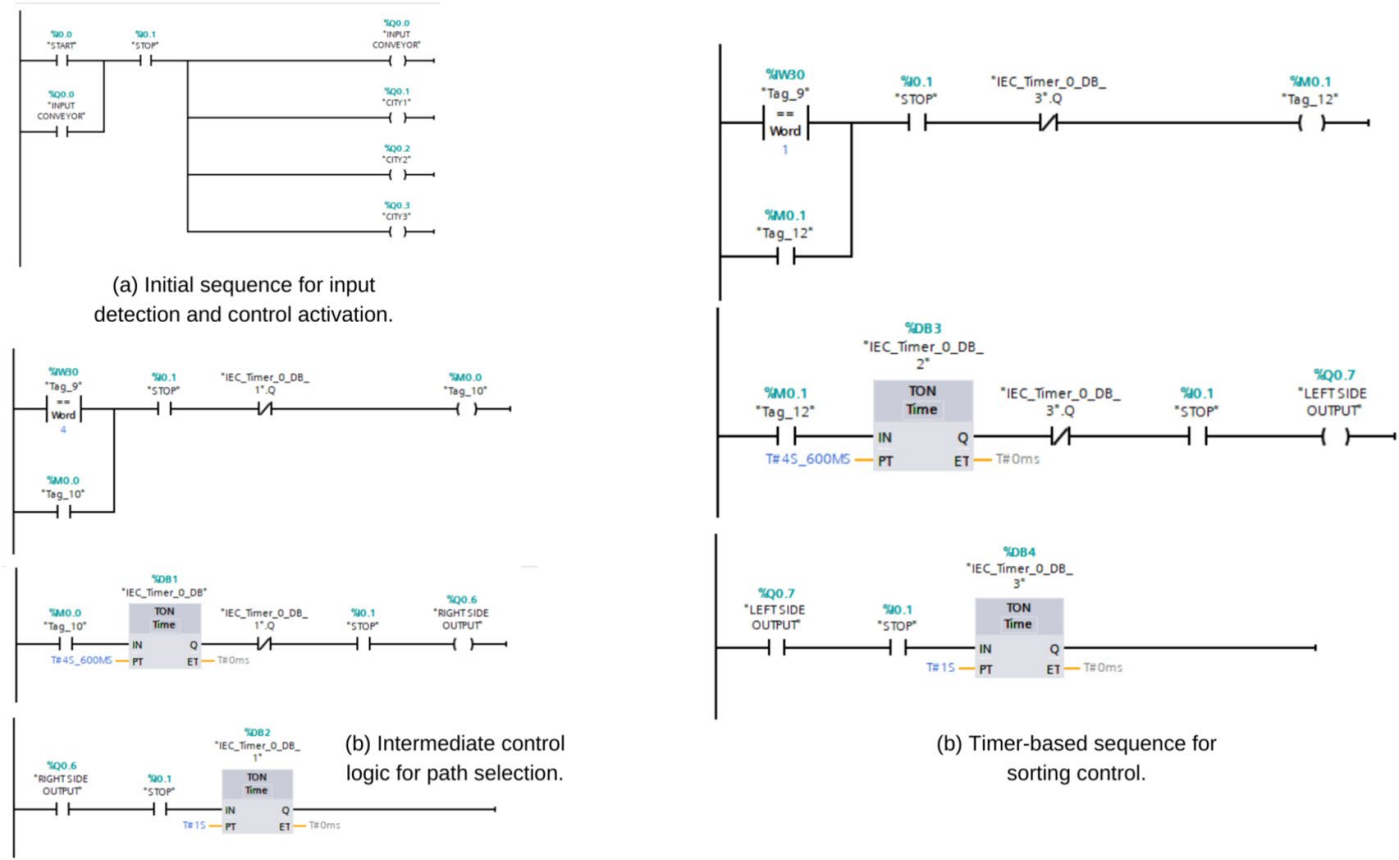
Ladder logic diagram showing input detection, timer-based control, path selection, and output activation.

Deflection was derived using:

3 $$\begin{aligned} EI \frac{d^2y}{dx^2} = M(x) \end{aligned}$$

where $E$ is the modulus of elasticity and $I$ is the moment of inertia. These calculations confirmed that the frame could handle operational loads with deflections remaining well within acceptable limits. Simulations were performed in Factory IO software to validate the functionality of the system under real-world conditions. Key parameters such as object speed, detection timing, and wheel activation were monitored. The simulation model was configured assuming ideal operational conditions, without environmental disturbances or mechanical losses, to isolate and validate logical correctness. The control logic for the sorting system was developed using ladder programming in a Siemens S7-1200 PLC, involving sequential processes for input detection, barcode evaluation, and output activation. The consolidated ladder diagram, shown in Fig. 4, illustrates the operational flow of the system. Initially, sensors and barcode scanners detect objects and transmit data to the PLC. Timers within the logic synchronize the activation of omni wheels, ensuring smooth and efficient operation. The barcode data are evaluated to determine the correct sorting path, and the corresponding omni wheel is activated to direct the object to its designated destination. This cascaded ladder diagram highlights the sequential processes, ensuring clarity and optimizing the system’s performance for high-speed sorting.

The given algorithm 1 was used that outlines the sequence of operations for the proposed ASS, from initialization to sorting based on barcode evaluation. This stepwise approach ensured clarity in describing the automation process.

**Algorithm 1:**
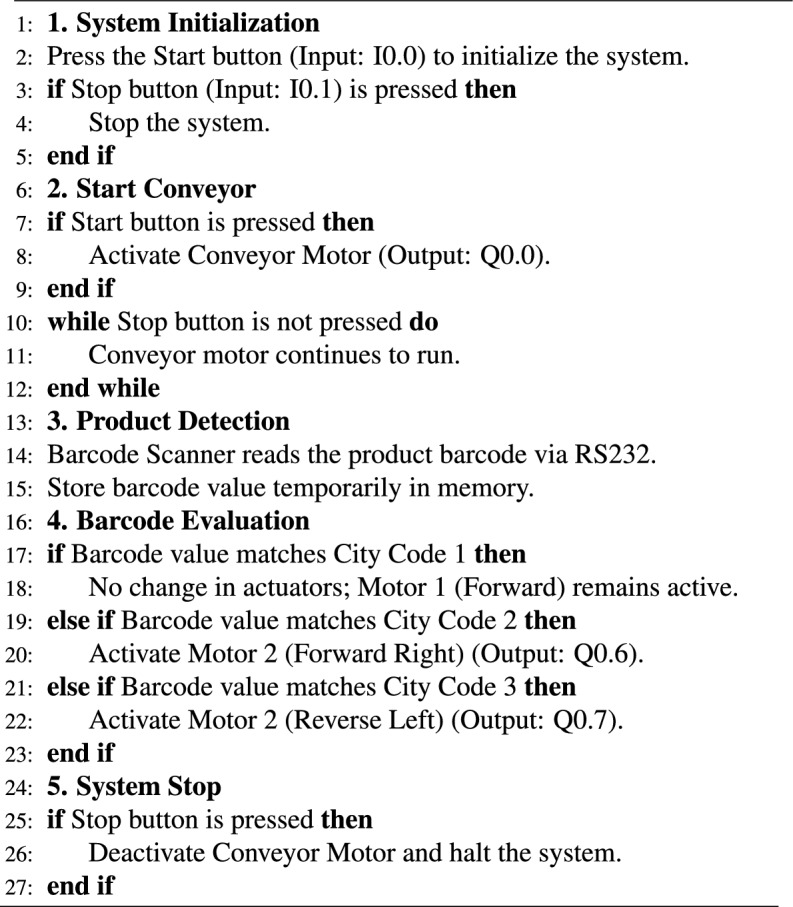
PLC-Controlled Conveyor Sorting System

**Fig. 5:**
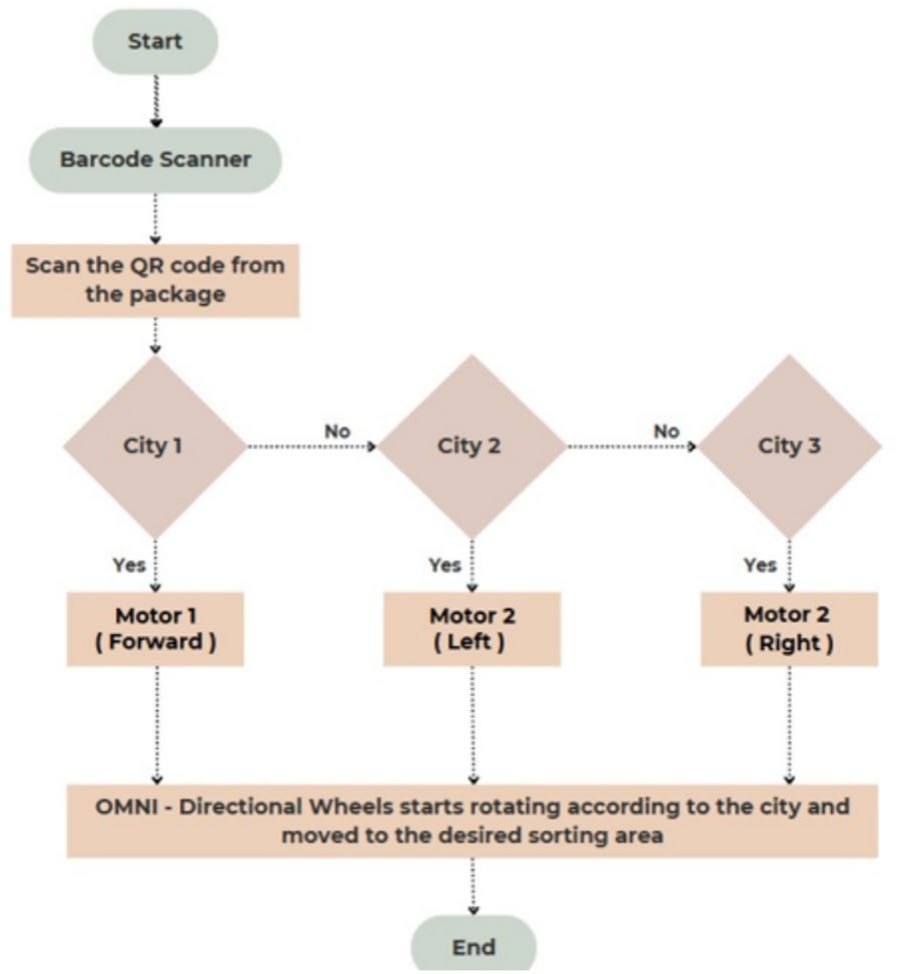
Flowchart illustrating the decision-making logic for barcode-based sorting using PLC-controlled omni wheels.

To further clarify the sorting process, a flowchart illustrating the barcode evaluation and motor activation logic has been added, as shown in Fig. 5. The flowchart demonstrates how the system evaluates the scanned barcode and activates the appropriate omni wheel motor to sort the object towards its designated location. City codes embedded in the barcode are mapped to predefined actuator states, allowing real-time object redirection.

Simulations for the proposed system were conducted using Factory I/O (Version 2.5.7), a 3D industrial automation simulation platform that facilitated the development and testing of the virtual sorting environment under idealised warehouse conditions. The control logic was programmed using Siemens Totally Integrated Automation (TIA) Portal (Version 17), which enabled ladder logic implementation for the Siemens S7-1200 PLC. To interface the simulation environment with the PLC program in real time, S7 PLCSIM was employed as a virtual link, allowing for accurate testing and verification of the control sequence. Together, these software tools supported an integrated workflow for modelling, programming, and virtual commissioning. Further information on Factory I/O can be accessed at https://factoryio.com.

## Results and discussion

### Mathematical analysis and calculations

As mentioned earlier, mathematical analysis was performed to validate the design of the proposed ASS, for structural integrity, roller speed calculations, and frame strength. These calculations ensure that the system is both operationally efficient and structurally sound.

#### Beam analysis: reactions and deflections

**Fig. 6:**
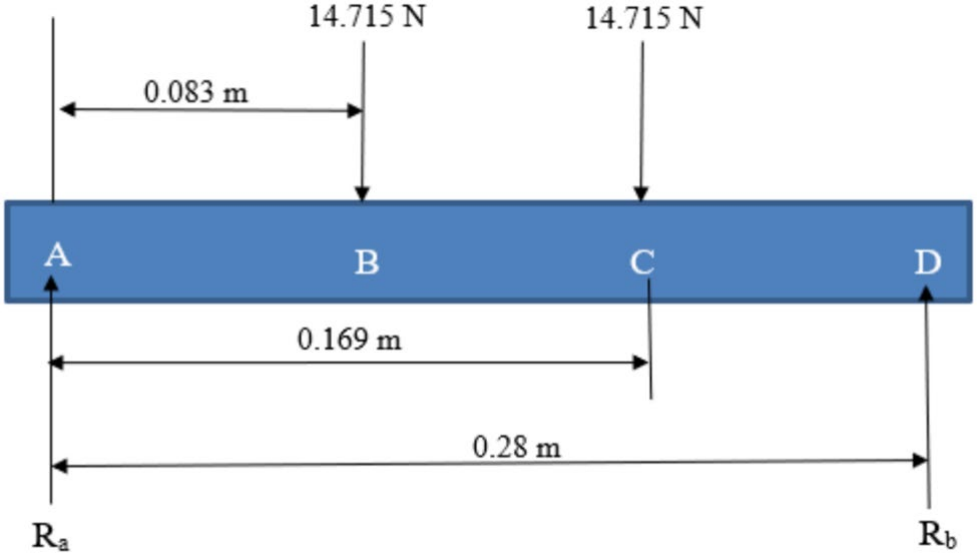
Beam diagram showing loading conditions and reaction forces.

The beam supporting the conveyor was analyzed under applied loads. Figure 6 illustrates the loading conditions, with two concentrated loads. The reactions at supports ($R_a$ and $R_b$) were calculated using equilibrium conditions.

Using vertical equilibrium ($\Sigma F_y = 0$):

4 $$\begin{aligned} R_a + R_b = 29.43 \, \text {N}. \end{aligned}$$

Moments about point $A$ ($\Sigma M_A = 0$) yield:

5 $$\begin{aligned} R_b = 13.23 \, \text {N}, \quad R_a = 16.2 \, \text {N}. \end{aligned}$$

The deflection of the beam was calculated using:

6 $$\begin{aligned} EI \cdot y = 16.2 \left( \frac{x^3}{6}\right) - 14.715 \left( \frac{(x - 0.083)^3}{6}\right) - 14.715 \left( \frac{(x - 0.169)^3}{6}\right) . \end{aligned}$$

Boundary conditions yielded deflections:

7 $$\begin{aligned} y_1 = 0.001068 \, \text {mm}, \quad y_2 = 0.00896 \, \text {mm}. \end{aligned}$$

#### Roller speed calculations

The conveyor speed and the roller speed were calculated to achieve a throughput of 2000 objects per hour, for a conveyor length of $0.3 \, \text {m}$:

8 $$\begin{aligned} \text {Conveyor speed} = \frac{600 \, \text {m}}{3600 \, \text {s}} = 0.167 \, \text {m/s}. \end{aligned}$$

The roller speed, based on a roller diameter of $0.09 \, \text {m}$, was calculated as:

9 $$\begin{aligned} \text {Roller speed (RPM)} = \frac{\text {Conveyor speed}}{\text {Roller circumference}} \cdot 60 = 35.46 \, \text {RPM}. \end{aligned}$$

#### Frame design calculations

The frame strength was evaluated to support a distributed load of $10 \, \text {kg}$. The load per leg was calculated as:

10 $$\begin{aligned} F_{\text {leg}} = \frac{F}{4} = 24.5 \, \text {N}. \end{aligned}$$

Using a yield strength of $69 \, \text {MPa}$ for aluminum, the required cross-sectional area was determined:

11 $$\begin{aligned} A = \frac{F_{\text {leg}}}{\sigma _y} = 0.355 \, \text {cm}^2. \end{aligned}$$

The actual cross-sectional area of the frame, assuming outer diameter $D = 3 \, \text {cm}$ and inner diameter $d = 1 \, \text {cm}$, was calculated as:

12 $$\begin{aligned} A = \frac{\pi }{4} (D^2 - d^2) = 0.62 \, \text {cm}^2. \end{aligned}$$

The calculations thus ensured that the frame design is adequate to withstand the applied load, with a significant safety margin.

### Simulation results

**Fig. 7:**
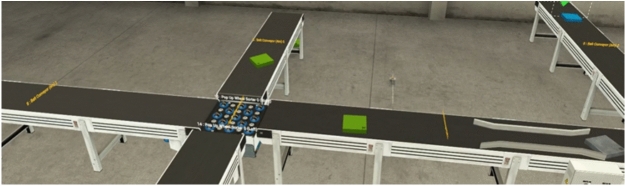
Factory IO simulation: Objects directed to sorting paths.

**Fig. 8:**
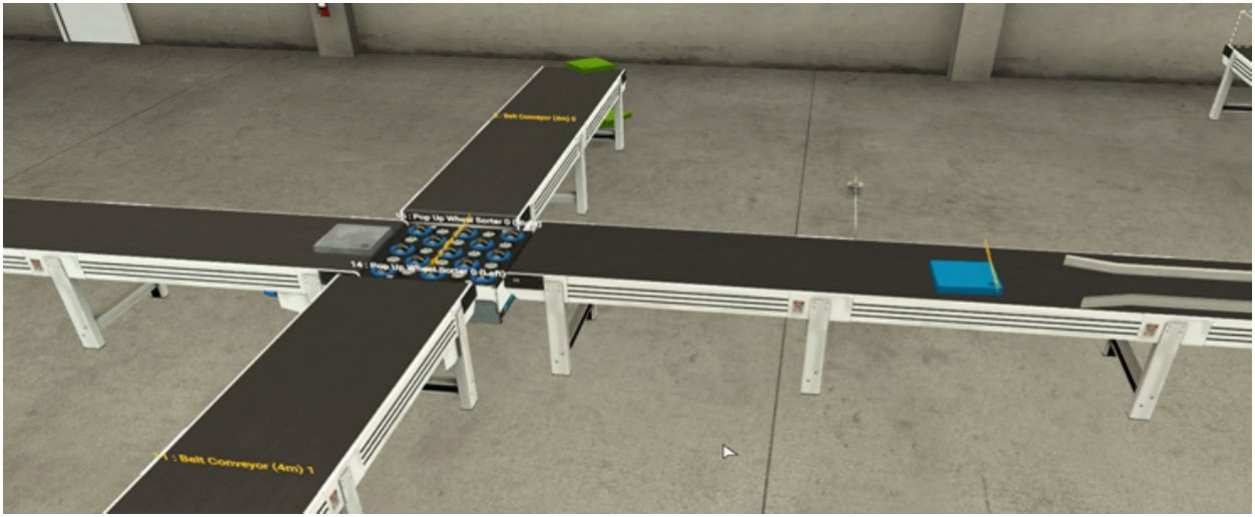
Factory IO simulation: Conveyor in operation during sorting.

The simulation of the proposed omnidirectional ASS, conducted in Factory I/O, validated its functionality. The key parameters included a conveyor speed of $0.167 \, \text {m/s}$, derived from mathematical analysis, and an assumed barcode detection accuracy of $100\%$. Standard object dimensions of $0.1 \, \text {m} \times 0.1 \, \text {m} \times 0.1 \, \text {m}$ were used, with a response delay of $100 \, \text {ms}$ configured between object detection and omni wheel activation. The ladder logic incorporated a timer delay to synchronize the conveyor’s movement and sorting operations. The Factory I/O environment modeled an idealized industrial warehouse setting to isolate the control logic’s performance. The following assumptions were made: (i) perfect barcode scanning with 100% accuracy and no latency, (ii) negligible conveyor friction or slippage, (iii) no environmental disturbances such as dust, vibration, or lighting inconsistencies, and (iv) uniform loading of objects without variation in mass or spacing. These conditions enabled a deterministic evaluation of the PLC logic, but real-world deployment would require additional validation under dynamic environmental conditions.The simulation showcased the sorting process, with objects being directed to different paths based on barcode data. Figures 7 and 8 illustrate the system in operation, demonstrating precise object handling by the PLC-controlled omni wheels.

**Table 1:** Summary of key simulation parameters and outcomes.

Parameter	Value/Assumption
Simulation environment	Factory I/O (Version 2.5.7) - idealised warehouse setting
Conveyor speed	$0.167 \, \text {m/s}$
Roller speed	$35.46 \, \text {RPM}$
Object dimensions	$0.1 \times 0.1 \times 0.1 \, \text {m}$
Barcode detection accuracy	100% (assumed)
Sensor-to-actuator response delay	$100 \, \text {ms}$
Friction and disturbances	Negligible / not simulated
Load condition	Uniform, $10 \, \text {kg}$ total
Sorting accuracy	$98\%$
Throughput	2000 objects per hour

The system achieved a sorting accuracy of approximately $98\%$ with a throughput of 2000 objects per hour at a conveyor speed of $0.167 \, \text {m/s}$,as summarised in Table 1. These results validate the integration of barcode-based classification with omni wheels and PLC control, enabling high-speed, flexible, and precise sorting. Compared to conventional systems such as pusher or tilt-tray mechanisms constrained by rigid layouts^19,20^, omni wheels provided multidirectional flexibility, enhancing adaptability and reducing response times. Although Zaher et al.^18^ demonstrated similar accuracy using reinforcement learning, their approach required high computational resources and complex setups. In contrast, this study leverages deterministic PLC logic, offering simplicity, cost-effectiveness, and reliability suitable for industrial applications. The multidirectional mobility of the omni wheels, facilitated by rollers perpendicular to the primary axis, minimizes lateral forces and friction, ensuring smooth transitions and precise directional control. This low-friction movement reduces energy losses, enabling faster response times for object redirection. The ladder logic implemented in the Siemens S7-1200 PLC synchronizes input signals from sensors and barcode scanners, optimizing timing and reducing delays. The beam analysis confirmed the structural integrity of the system, with deflections well within the permitted limits ($y_{\text {max}} = 0.00896 \, \text {mm}$), ensuring stable performance under varying loads. The calculated roller speed of $35.46 \, \text {RPM}$ met throughput requirements, demonstrating efficient power transmission and consistent material flow. Thus, this study addressed the gaps identified in previous research by combining barcode-based classification, omni wheels, and PLC-controlled logic into a unified system. The results highlight the system’s ability to achieve high-speed, accurate sorting with scalability and adaptability. Despite its advantages, the system has limitations that warrant further investigation. Future research should focus on real-world testing under dynamic environmental conditions, incorporating advanced sensors, load variability, and IoT-enabled monitoring to ensure robustness. Hybrid control strategies combining PLC logic with machine learning may further enhance the system’s adaptability and fault tolerance for large-scale industrial deployment.

## Conclusion

This work demonstrates the development of a PLC-controlled omni wheel conveyor sorting system that integrates barcode-based classification with high-speed and precise sorting capabilities. The proposed system achieves a throughput of 2000 objects per hour with a sorting accuracy of $98\%$, validated through simulations in Factory IO. Omni wheels enhanced flexibility and reduced energy losses, while deterministic ladder logic ensured synchronized and reliable operations. The beam analysis confirmed structural integrity and the roller speed calculations aligned with the demands of throughput. Compared to existing methods, the system offers a simpler and more cost-effective alternative suitable for industrial applications. Despite its advantages, limitations such as idealized simulation conditions and untested scalability highlight areas for future research. Addressing these challenges through IoT integration, dynamic adaptability, and hybrid control strategies could further enhance the system’s performance and applicability in diverse industrial settings. This study provides a solid foundation for the advancement of automated sorting systems in the material handling industries. Although the present study is limited to virtual commissioning through Factory I/O simulations, future phases of the research will focus on identifying suitable industry partners for real-time implementation. The system will be deployed in select logistics or warehouse environments to evaluate its robustness under actual operating conditions, including variable loading, sensor response delays, and ambient disturbances. These real-world validations will help assess scalability and fine-tune control logic, further strengthening the system’s industrial readiness. As such, this work serves as a foundational step toward full-scale deployment and long-term adoption in the material handling sector.

## Data Availability

The datasets generated and/or analyzed during the current study are available from the corresponding author on reasonable request.
